# Extended-spectrum beta-lactamase- and carbapenemase-producing *Escherichia coli* isolates causing hospital- and community-acquired infections in Tunisia (2001–2019): expansion of CTX-M-15-C2 and CTX-M-27-C1 ST131 subclades

**DOI:** 10.1128/spectrum.01471-24

**Published:** 2024-10-25

**Authors:** Nesrine Sallem, Noura Ben Mansour, Hana Amri, Mohamed Boudaoura, Olfa Gargouri, Faouzia Mahjoubi, Adnene Hammami, Basma Mnif

**Affiliations:** 1 Laboratory of Microbiology, Research Laboratory for Microorganisms and Human Disease, Habib Bourguiba University Hospital, University of Sfax, Sfax, Tunisia; 2 Regional Hospital Of Jebeniana, Sfax, Tunisia; 3 Policlinique de la Caisse Nationale de Sécurité Sociale, Sfax, Tunisia; MultiCare Health System, Tacoma, Washington, USA

**Keywords:** ESBL, *Escherichia coli*, carbapenemases, ST131

## Abstract

The prevalence of infections caused by extended-spectrum beta-lactamase (ESBL)-producing *Escherichia coli* (ESBL-EC) and carbapenemase-producing *E. coli* (CP-EC) is increasing worldwide. We investigated the epidemiology of ESBL-EC and CP-EC causing hospital-acquired (HA) infections in a large teaching hospital in Tunisia over the last two decades and compared it with a collection of 107 community-acquired (CA) ESBL-EC isolates. Between 2001 and 2019, the incidence of HA ESBL-EC increased significantly from 0.08 to 0.32 cases per 1,000 patient days, due entirely to the rapid emergence and expansion of ST131, which accounted for 42.3% (157/371) of HA ESBL-EC. Most ESBL-EC harbored the CTX-M type (92%) with a predominance of blaCTX-M-15. The C2/H30-Rx subclone (*n* = 103, 65.6%) accounted for 90% of ST131 isolates between 2003 and 2012 and was exclusively associated with CTX-M-15, whereas cluster C1-M27, which was associated with CTX-M-27, emerged in 2013 and expanded gradually to 55% of ST131 in 2019. ST131 prevalence was higher among CA ESBL-EC than HA ESBL-EC (63.6% vs. 42.3%, *P* = 0.002). CA C2 subclone and non-ST131 isolates showed higher virulence scores than HA isolates. The incidence of CP-EC remained stable over the study period with a mean of 0.08 cases per 1,000 patient days. Among the 38 identified CP-EC isolates, only 16.2% belonged to the ST131 clone and 81.5% produced OXA-48-like carbapenemases. ST131 is the major driver of ESBL-EC spread in both hospital and community settings in Tunisia, mainly linked to the expansion of the CTX-M-15-C2 and CTX-M-27-C1 subclades. The emergence of CP-EC requires ongoing genomic surveillance.

**IMPORTANCE:**

We aimed to investigate the microbiological features of extended-spectrum beta-lactamase (ESBL)-producing *Escherichia coli* (ESBL-EC) and carbapenemase-producing *E. coli* (CP-EC) causing hospital- and community-acquired infections in Tunisia over the last two decades. The study captured the emergence and expansion of the CTX-M-15-C2 ST131 subclade and successively the CTX-M-27-C1 ST131 subclade, which were responsible for the steady increase in the prevalence of ESBL-EC. However, the incidence of CP-EC remained stable over the study period with a highly diverse content in carbapenemase genes dominated by blaOXA-48-like. This is the first study to provide comprehensive data on the epidemiology of ESBL-EC and CP-EC in a North African country.

## INTRODUCTION

Extraintestinal pathogenic *Escherichia coli* (ExPEC) is the leading cause of both community-acquired and hospital-acquired (HA) Gram-negative infections. The incidence of these infections is increasing worldwide (1). Furthermore, cephalosporin and carbapenem resistance mediated by extended-spectrum β-lactamases (ESBLs) and carbapenemases, respectively, is increasing worldwide, in both healthcare and community settings, and such resistance is compromising the antibiotic treatment options of *E. coli* infections (2).

Epidemiological studies of ExPEC from all continents have demonstrated that four sequence types (ST), including ST131, ST73, and ST95 belonging to phylogroup B2, and ST69 belonging to phylogroup D, represent more than 50% of the isolates (1, 3). ST131 is the most prevalent *E. coli* clone isolated in human infections. Its success was due to its rapid global dissemination since its emergence in the 2000 s and its frequent multidrug resistance and virulence (4, 5). ST131 is composed of three different clades: A, B, and C. Clades A and B are generally minor clades with less resistance. However, clade C is the largest clade and comprises two subclades that are resistant to fluoroquinolones: C1/H30R and C2/H30Rx. Subclade C2, which is tightly associated with CTX-M-15, is the most successful and expanded ST131 sublineage and is largely responsible for the global increase of CTX-M-15 ESBLs (6–8). Whereas, subclade C1-M27, associated with CTX-M-27, has recently expanded in Japan, then in many other countries (8, 9). ST131 has already been detected in Tunisia among extended-spectrum beta-lactamase (ESBL)-producing *E. coli* (ESBL-EC). However, the prevalence of ST131 among unselected *E. coli* isolates and the epidemiology of its subclades in Tunisia remain unknown (10–12). Other important *E. coli* clones have been detected among ESBL-EC including ST405, ST38, ST648, ST410, and ST1193, which is an emerging multidrug high-risk clone imitating *E. coli* ST131 (8, 13).

As ST131 is now frequently encountered in carbapenemase-producing *E. coli* (CP-EC), the ST131-CP-EC might follow the same expansion and dissemination in hospital and community settings as the ST131-CTX-M scenario (8). Nevertheless, the epidemiology of CP-EC seems to be more complex than that of ESBL-EC with a broad geographical diversity in terms of carbapenemase genes and dominant lineages (14).

Comprehensive epidemiology data about ESBL-EC and CP-EC are limited to small studies in Tunisia (11, 15). However, large surveillance studies monitoring the expansion of antimicrobial resistance and high-risk clones are required to implement prevention strategies. This study aimed to characterize the epidemiology of ESBL-EC and CP-EC causing HA infections in a large teaching hospital in Tunisia over the last two decades (2001–2019) and to compare the population structure of hospital-acquired ESBL-EC (HA ESBL-EC) and community-acquired ESBL-EC isolates. We investigated trends in ST131 prevalence and captured the introduction of ST131 subclades.

## RESULTS

### Hospital-acquired ESBL-EC

**Fig 1:**
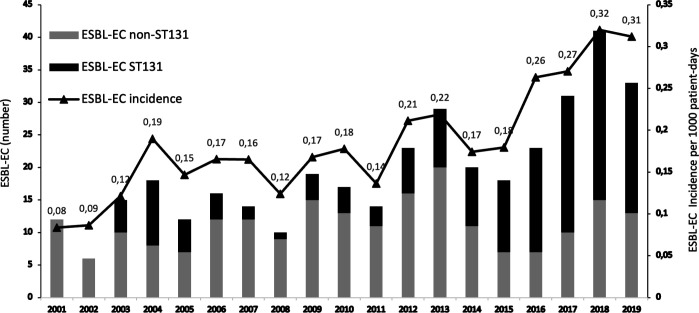
Annual incidence of ESBL-EC isolates and prevalence of ESBL-EC ST131 and ESBL-EC non-ST131 among tested isolates in Habib Bourguiba Hospital, 2001–2019.

Between 2001 and 2019, a total of 6,792 non-duplicate *E. coli* were isolated from inpatients/outpatients in Habib Bourguiba University Hospital (HBUH) of Sfax, Tunisia. The prevalence of ESBL-EC was 18.94% and increased significantly from 6.31% in 2001 to 29.55 % in 2019. A total of 488 non-duplicate isolates met the definition of HA ESBL-EC. The incidence of HA ESBL-EC increased from 0.08 cases per 1,000 patient days in 2001 to 0.31 cases per 1,000 patient days in 2019 (*P* = 0.01; Fig. 1).

**Fig 2:**
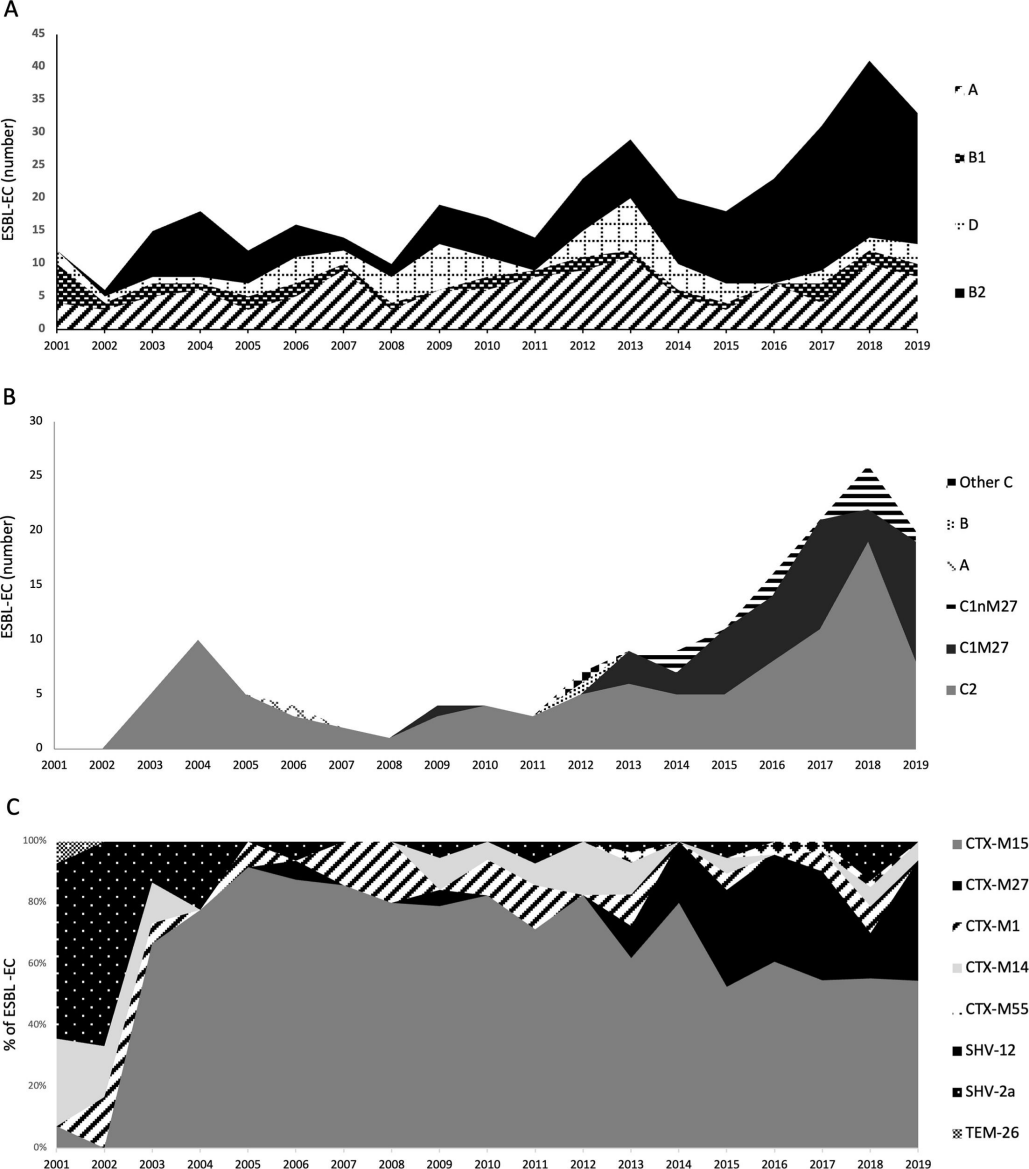
Temporal distribution of HA ESBL-EC isolates and ESBL types at Habib Bourguiba Hospital (2001 to 2019). (**A**) Temporal distribution of *E. coli* phylogroups; (**B**) temporal distribution of *E. coli* ST131 clonal groups. (**C**) Temporal distribution of ESBL types as a proportion of all ESBL-EC. The x-axis represents the isolation year. Shading differences reflect the presence of each phylogroup or ST131 clone. The y-axis represents the number of episodes caused by each phylogroup (**A**) and the percentage of a given ST131 clone (**B**) or an ESBL type (**C**).

Only 371 HA ESBL-EC isolates had been stored and were available for further analysis. They were predominantly isolated from urine (*n* = 199; 53.6%); other clinical sites were abdominal deep infections (*n* = 53), blood (*n* = 40), pulmonary (*n* = 38), wounds (*n* = 34), and others fluids (*n* = 7) (Table S1). The large majority (350/380, 92.1%) of the ESBLs produced were of the CTX-M group, with 247 (65%) CTX-M-15, 54 (14.2 %) CTX-M-27, 24 (6.3%) CTX-M-14, 21 (5.5%) CTX-M-1, and 4 (1%) CTX-M-55. Only 23 (6%) SHV-12, 6 (1.6 %) SHV-2a, and 1 (0.3 %) TEM-26 were detected, principally in the early 2000 s. The most important progression concerned CTX-M-15 and CTX-M-27 (Fig. 2). Nine (2.4 %) strains harbored two ESBL genes: *bla*_CTX-M_ and *bla*_SHV_.

Resistance rates were high for fluoroquinolones (85.4%) and gentamicin (59.3%) and low for imipenem (5.1%), nitrofurantoin (3.5%), and fosfomycin (1.3%). Nineteen isolates produced different carbapenemases: 13 OXA-48-like, 5 NDM, and 1 VIM. 202 (54.4%) ESBL-EC isolates were positive for *aac(6′)-lb-cr* and 16 (4.3 %) for *qnr* genes. Only two isolates were positive for *mcr-1* with colistin MIC values of 8 mg/L and 16 mg/L and one for *fosA3* gene with fosfomycin MIC > 32 mg/L (Table 1).

**TABLE 1:** Molecular characteristics and antibiotic susceptibility of the HA ESBL *E. coli* isolates according to ST131 clades

	No. (%) of HA ESBL-EC isolates						*P* value	*P* value
	ST131 C2 (103)	ST131 C1M27 (42)	ST131 C1nM27 (9)	ST131 others (3)	ST131 (157)	Non ST131 (214)	C1-M27 vs. C2	ST131 vs. non-ST131
Virulence factors								
Adhesin								
*fimH*	103 (100)	42 (100)	9 (100)	3 (100)	157 (100)	183 (85.5)		0.0001
*papAH*	45 (43.7)	0	1	0	46 (29.3)	38 (17.8)	<0.00001	<0.00001
*papC*	42 (40.8)	20 (47.6)	4	2	68 (43.3)	25 (11.7)		<0.00001
*papEF*	36 (35)	0	0	0	36 (22.9)	35 (16.4)	<0.00001	
*papGI*	0	0	0	0	0	2 (0.9)		
*papGII-III*	45 (43.7)	0	0	0	45 (28.7)	21 (9.8)	<0.00001	<0.00001
*sfa/focDE*	0	0	0	0	0	35 (16.4)		<0.00001
*afa/draBC*	7 (6.8)	0	0	0	7 (4.5)	7 (3.3)		
*yfcV*	103 (100)	42 (100)	9	3	157 (100)	43 (20.1)		<0.00001
Toxin								
*sat*	97 (94.2)	42 (100)	7	1	147 (93.6)	56 (26.2)		<0.00001
*cnf1*	45 (43.7)	0	0	0	45 (28.7)	12 (5.6)	<0.00001	<0.00001
*hlyA*	49 (47.6)	0	0	0	49 (31.2)	13 (6.1)	<0.00001	<0.00001
Iron uptake								
*iutA*	103 (100)	41 (97.6)	7	3	154 (98.1)	129 (60.3)		<0.00001
*iroN*	7 (6.8)	0	0	0	7 (4.5)	33 (15.4)		0.0014
*fyuA*	103 (100)	42 (100)	9	3	157 (100)	102 (47.7)		<0.00001
*chuA*	103 (100)	42 (100)	9	3	157 (100)	53 (24.8)		<0.00001
Capsule								
*kpsM II*	99 (96.1)	42 (100)	9	3	153 (97.5)	54 (25.2)		<0.00001
*kpsM II-K5*	42 (40.8)	40 (95.2)	9	0	91 (58)	33 (15.4)	<0.00001	<0.00001
*kpsM III*	5 (4.9)	0	0	0	5 (3.2)	21 (9.8)		0.0235
Miscellaneous								
*irp2*	103 (100)	42 (100)	8	3	156 (99.4)	110 (51.4)		<0.00001
*ear*	97 (94.2)	41 (97.6)	8	2	148 (94.3)	122 (57)		<0.00001
*cvaC*	4 (3.9)	0	1	0	5 (3.2)	12 (5.6)		
*hra*	56 (54.4)	4 (9.5)	2	0	62 (39.5)	47 (22)	<0.00001	0.0003
*pher*	55 (53.4)	41 (97.6)	8	3	107 (68.2)	157 (73.4)	<0.00001	
*traT*	86 (83.5)	18 (42.9)	9	1	114 (72.6)	149 (69.6)	<0.00001	
*ibeA*	1 (1)	1 (2.4)	0	1	3 (1.9)	3 (1.4)		
*malX*	103 (100)	42 (100)	9	3	157 (100)	38 (17.8)		<0.00001
*usp*	103 (100)	42 (100)	9	3	157 (100)	29 (13.6)		<0.00001
Mean per isolate	15.94	13.90	13.11	11.33	15.2	6.44	<0.0001	<0.00001
ExPEC^*a*^	101 (98.1)	41 (97.6)	7	3	152 (96.8)	58 (27.1)		<0.00001
Virotype								
A	6 (5.8)	0	0	0	6 (3.8)	–		
B	3 (2.9)	0	0	0	3 (1.9)	–		
C	40 (38.8)	41 (97.6)	7	1	89 (56.7)	–	<0.00001	
D	1 (1)	1 (2.4)	0	1	3 (1.9)	–		
E	43 (41.7)	0	0	0	43 (27.4)	–	<0.00001	
F	3 (2.9)	0	0	0	3 (1.9)			
IND	7 (6.8)	0	2	1	10 (6.4)	–		
Resistance genes								
*bla*_CTX-M-15_	103 (100)	0	0	2	105 (66.9)	142 (66.4)	<0.00001	
*bla*_CTX-M-27_	0	42 (100)	7	1	50 (31.8)	4 (1.8)	<0.00001	<0.00001
*bla*_CTX-M-14_	0	0	0	0	0	24 (11.2)		<0.0001
*bla*_CTX-M-1_	0	0	0	0	0	21 (9.8)		0.0001
*bla*_CTX-M-55_	0	0	0	0	0	4 (1.8)		
*bla*_SHV-12_	5	0	0	0	5 (3.2)	18 (8.4)		0.0004
*bla*_SHV-2a_	0	0	0	0	0	6 (2.8)		
*bla*_TEM-26_	0	0	0	0	0	1 (0.5)		
*bla*_OXA-48_	1 (1)	0	0	0	1 (0.6)	7 (3.3)		
*bla*_OXA-204_	3 (2.9)	0	0	0	3 (1.9)	1 (0.5)		
*bla*_OXA-181_	0	0	0	0	0	1 (0.5)		
*bla*_NDM-1_	1 (1)	0	1	0	2 (1.3)	3 (1.4)		
*bla*_VIM-2_	0	0	0	0	0	1 (0.5)		
*aac(6′)-Ib-cr*	102 (99)	1 (2.4)	0	0	103 (65.6)	99 (46.3)	<0.00001	0.0003
*qnrA*	0	1 (2.4)	0	1	2 (1.3)	2 (0.9)		
*qnrB*	0	1 (2.4)	0	0	1 (0.6)	11 (5.1)		0.0335
*qnrS*	0	0	0	0	0	8 (3.7)		0.0368
*aqxAB*	0	0	0	0	0	2 (0.9)		
*mcr-1*	0	0	0	0	0	2 (0.9)		
*fosA3*	0	0	0	0	0	1 (0.5)		
Antibiotics								
Amoxicillin-clavulanate	95 (92.2)	7 (16.7)	6	1	109 (69.4)	171 (79.9)	<0.00001	0.0281
Imipenem	5 (4.9)	0	1	0	6 (3.8)	13 (6.1)		
Amikacin	32 (31.1)	1 (2.4)	1	0	34 (21.7)	30 (14)	0.0004	
Gentamicin	75 (72.8)	1 (2.4)	2	0	78 (49.7)	142 (66.4)	<0.00001	0.0017
Tobramycin	97 (94.2)	1 (2.4)	3	0	101 (64.3)	160 (74.8)	<0.00001	0.0394
Ciprofloxacin	103 (100)	42 (100)	9	1	155 (98.7)	162 (75.7)		<0.00001
Trimethoprim/sulfamethoxazole	73 (70.9)	38 (90.5)	4	3	118 (75.2)	159 (74.3)	0.0208	
Fosfomycin	0	1 (2.4)	1	0	2 (1.3)	3 (1.4)		
Addiction system								
*ccdAB*	90 (87.4)	42 (100)	9	3	144 (91.7)	136 (63.6)	0.0363	<0.00001
*pemKI*	85 (82.5)	41 (97.6)	9	2	137 (87.3)	154 (72)	0.0298	0.0006
*srnCB*	69 (67)	37 (88.1)	5	2	113 (72)	128 (59.8)	0.0167	0.0205
*hok/sok*	31 (30.1)	9 (21.4)	2	0	42 (26.8)	65 (30.4)		
*pndAC*	15 (14.6)	2 (4.8)	0	0	17 (10.8)	24 (11.2)		
*vagCD*	102 (99)	0	2	1	105 (66.9)	110 (51.4)	<0.00001	0.0040
*yacAB*	11 (10.7)	5 (11.9)	1	1	18 (11.5)	45 (21)		0.0223
*relEB*	3 (2.9)	0	0	0	3 (1.9)	5 (2.3)		
Mean per isolate	3.94	3.24	3.11	3	3.69	3.12	0.0078	0.0001

^*a*^ Isolates were categorized as extra-intestinal pathogenic *E. coli* (ExPEC) if more than two of five virulence factors, including *papAH* and/or *papC*, sfa/*focDE*, afa/*draBC*, *kpsM II*, and *iutA*, were detected.

^*b*^ (-) not applicable

One hundred and seventy-two (46.4%) isolates belonged to phylogroup B2, 115 (31%) to phylogroup A, 53 (14.3%) to phylogroup D, and 31 (8.4%) to phylogroup B1. The trends of the phylogroups did not significantly change during the study period except for phylogroup B2, which increased over the study period coinciding with the expansion of the ST131 clone (Fig. 2). The ST131 clonal group (*n* = 157) represented 91.3% of the B2 phylogroup and 42.3% of all ESBL-EC. The first ST131 isolate (C2-H30-Rx) was recovered in 2003. The ST131 prevalence among ESBL-EC increased substantially from 0% in 2001 to over 60% between 2015 and 2019 (Fig. 1). Among the 15 B2-non ST131 isolates, two were ST1193, one ST95, and one ST73. Four of the 53 group D isolates (7.5%) belonged to ST69. The expansion of ST131 was correlated with the increase in the overall incidence of ESBL-EC in our setting. However, it was not responsible for the decrease in the prevalence of Non-ST131 isolates as its incidence remained stable over time (*P* > 0.05).

Most ST131 isolates belonged to clade C (*n* = 155, 98.7%) with the following subclades: C2 (H30-Rx) (*n* = 103, 65.6%), C1-M27 cluster, (*n* = 42, 26.8%), C1-non-M27 cluster (*n* = 9, 5.7%), and one isolate belonging to other clade C. The two remainder ST131 isolates belonged to clade A and clade B. The prevalence of C2 subclade dominated the population structure of ST131 between 2003 and 2012 accounting for 90% of the ST131 population and was tightly associated with CTX-M15. However, since 2013, the prevalence of C1-M27 subclade has increased substantially from 33.3% of the total ST131 population in 2013 to 55% in 2019 (Fig. 2). There was a significant association between the expansion of the C1-M27 subclade and the increase in the CTX-M-27 prevalence among ESBL as 50 CTX-M-27 were reported within the ST131 isolates, precisely the C1 clade, versus two within the non-ST131 isolates (*P* < 0.00001).

Table 1 shows associations between ST131 subclades, ESBL types, and main virulence and resistance traits. The ST131 isolates exhibited a higher virulence score (mean 15.2) compared with the non-ST131 isolates (mean 6.44) (*P* < 0.001) as well as higher antibiotic resistance rates (aminoglycosides and fluoroquinolones) and plasmid addiction score (Table 1). The prevalence of ExPEC status (96.8 vs. 41.8%) (*P* < 0.001) was higher within ST131 isolates than within non-ST131 isolates (Table 1). The virulence factors (VFs) were distributed significantly by ST131 subclades. The prevalence of VFs, addiction systems, and antibiotic resistance rates were significantly higher within C2 subclade isolates than within C1-M27 subclade isolates (Table 1). The genes *papAH*, *papEF*, papGII, *cnf1*, *hlyA*, *aac(6′)-Ib-cr*, *vagCD*, and the virotype E were significantly associated with the C2 subclade isolates (Table 1).

### Hospital-acquired CP-EC

During the study period, 37 non-duplicate CP-EC were isolated, 19 of which coproduced ESBLs: 21 OXA-48, 6 OXA-204, 2 OXA-244, 1 OXA-181, 6 NDM-1, and one VIM-2. The first CP-EC isolate was an OXA-48 producer recovered in 2010 (Table 2; Table S2). The incidence of CP-EC was globally stable with a mean of 0.08 cases per 1,000 patient days. Almost half of the isolates belonged to phylogroup A (51.4%). Only six isolates (16.2%) belonged to ST131, of which five were C2 and one C1nM27 (Table 2). Two were OXA-244-producing ST38 isolates, and one was an NDM-producing ST1193. None belonged to the ST95, ST73, or ST69 clonal group. Of note, one phylogroup D isolate (ST1171, data not shown) coproducing *bla*_OXA-48_ and *mcr*-1 genes was recovered in 2018. The VF scores did not differ between CP-EC and ESBL-EC (Table S3).

**TABLE 2:** Characteristics of the CP-EC isolates

No. (%) of CP-EC isolates											
	2010	2011	2012	2013	2014	2015	2016	2017	2018	2019	Total
CP-EC isolates	3	2	4	7	3	2	6	2	5	3	37
CP-EC incidence (/1,000 patient days)	0.022	0.014	0.027	0.049	0.022	0.022	0.045	0.015	0.038	0.022	0.028
Carbapenemase type											
OXA-48	2	1	4	4	2		5	1	2		21 (56.7)
OXA-204	1	1		3					1		6 (16.2)
OXA-244									1	1	2 (5.4)
OXA-181							1				1 (2.7)
NDM-1					1	1		1	1	2	6 (16.2)
VIM-4						1					1 (2.7)
Associated ESBL	2	1	1	5	2	2	3	1	1	1	19 (51.4)
ESBL type											
CTX-M-15	2	1		4	1	2	3	1	1	1	16
CTX-M-14			1	1							2
CTX-M-27					1						1
Phylogenetic group											
A	1	2	3	2	2	1	4	1	2	1	19 (51.4)
B1				2		1				1	4 (10.8)
B2				3	1		1		1	1	7 (18.9)
D	2		1				1	1	2		7 (18.9)
ST131 isolates ST131 clade											6 (16.2)
C2				3			1		1		5
C1nM27					1						1

### Community-acquired ESBL-EC

One hundred and seven community-acquired ESBL-EC (CA ESBL-EC) were recovered between 2011 and 2016 mainly from urinary tract infections (93%) (Table 3). Unlike the HA ESBL-EC, the majority of the community-acquired isolates belonged to phylogroup B2 (74, 69.2% vs. 172, 46.4%, *P* < 0.0001), followed by phylogroup D (16, 15% vs. 53, 14.3%, *P* = 0.9864), A (12, 11.2% vs. 115, 31%, *P* <0.0001), and B1 (5, 4.7% vs. 31, 8.4%, *P* = 0.2874). Sixty-eight (63.6%) isolates were ST131, and one was ST73.

**TABLE 3:** Characteristics of the community-acquired ESBL-producing *E. coli* (CA-ESBL-EC) isolates

No. (%) of CA-ESBL-EC isolates							
	2011	2012	2013	2014	2015	2016	Total
ESBL isolates	3	12	6	18	40	28	107
ESBL type							
CTX-M-15	1	8	4	17	26	18	74 (66.1)
CTX-M-1		1	1		1	1	4 (3.6)
CTX-M-14	2	2	1		2	1	8 (7.1)
CTX-M-27				1	11	8	20 (17.9)
SHV-12		1		3	1	1	6 (4.5)
SHV-2a		1					1 (0.9)
Phylogenetic group							
A		4		1	4	3	12 (11.2)
B1		1		2	2		5 (4.7)
B2	1	4	2	15	30	22	74 (69.2)
D	2	3	4		4	3	16 (15)
ST131 isolates		4 (33.3)	2 (33.3)	14 (77.8)	26 (65)	22 (78.6)	68 (63.6)
ST131 clade							
C2		4	2	13	15	16	50 (73.5)
C1M27				1	3	4	8 (11.8)
C1nM27					6	2	8 (11.8)
A					1		1 (1.5)
B					1		1 (1.5)

The ST131 proportion among ESBL-E was significantly higher in community-acquired infections than in HA infections (63.6% vs. 42.3%, *P* = 0.002), even within the same period, 2011–2016 (63.6% vs. 43.3%, *P* = 0.00309).

Subclade C2 was the commonest subclade detected among the 68 ST131 isolates (50 isolates; 73.5%), followed by cluster C1-M27 and cluster non-C1-M27 (8 isolates each; 11.8%), and clade A and B (1 isolate each). The C1-M27 subclade was first detected in 2014 in the community setting but remained less prevalent than that in the hospital setting (11.8% vs. 26.8%, *P* = 0.0164)

The VFs scores were significantly higher among CA-ESBL-EC than among HA-ESBL-EC for both ST131 and non-ST13 isolates. Even with stratification by ST131 subclades, the VF scores remained significantly higher among the community-acquired subclade C2 isolates (18.2 vs. 15.42, *P* < 0.0001). However, HA-non-ST131 isolates showed higher resistance rates to antibiotics, especially aminoglycosides and fluoroquinolones, and were richer in addiction systems, especially *pemKI* and *vagCD*, than CA-non-ST131 isolates (Table S4).

**Fig 3:**
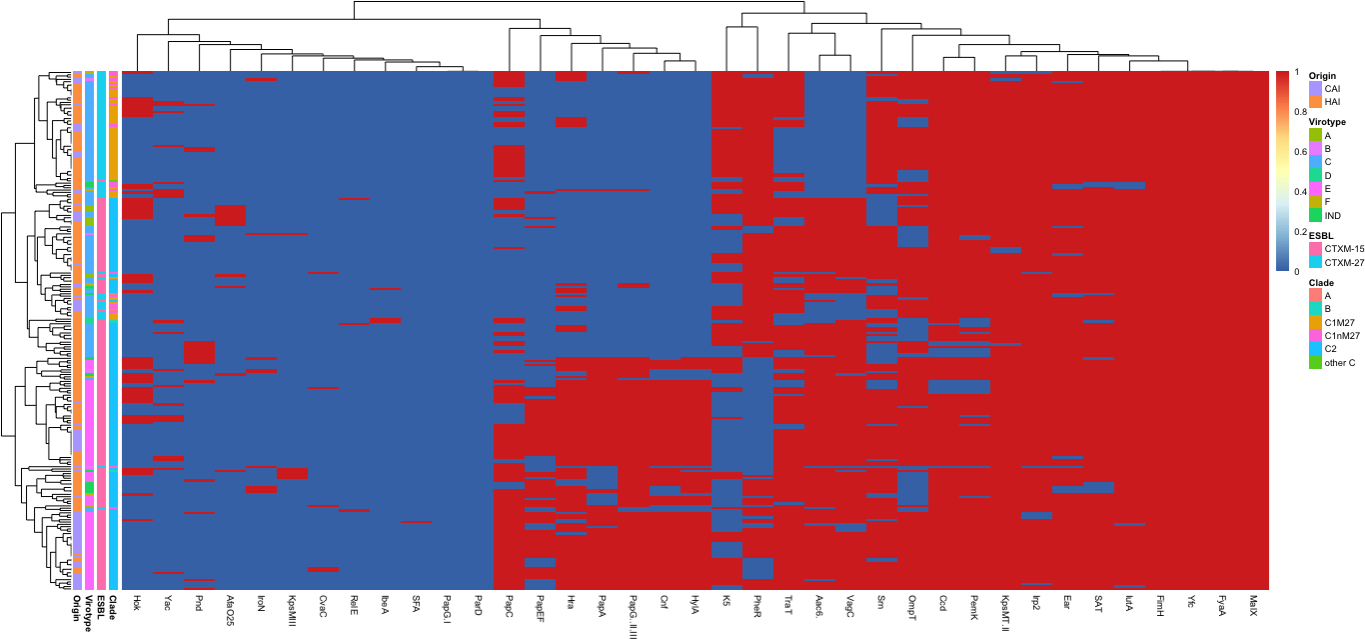
Heatmap plot demonstrating the pattern of VF and addiction system distribution across ST131 isolates according to their origin, virotype, ESBL type, and clade. Red represents the presence, and blue represents the absence of a VF or addiction system (CAI, community-acquired infection; HAI, hospital-acquired infection).

Overall, regardless of the onset origin of the infection, 96% of all the 225 ST131 studied isolates qualified as ExPEC. Although the majority of ST131 studied isolates belonged to virotype C (57.5%, 107/225) and virotype E (37.3%, 84/225) and accordingly clustered into two large groups as shown by the heatmap, a highly diverse virulence and addiction system gene content was observed (Fig. 3). Indeed, the 28 studied VFs and the 9 PAS occurred in 157 distinct combinations (157 profiles; Fig. 3).

## DISCUSSION

This work, one of the very few long-term surveillance studies—and the first in Tunisia—to analyze the dynamics of ESBL-EC and C-EC isolated from hospital and community-acquired infections, supports three main conclusions: (i) HA ESBL-EC prevalence increased over time in this university hospital, mainly driven by a continuous expansion of the ST131 subclades, (ii) ST131 prevalence was higher in the community setting, and (iii) we captured the emergence of C-EC but did not note their expansion in the hospital setting.

In this study of ESBL-EC spanning 19 years in a Tunisian university hospital, we found ESBL prevalence to increase at least four times reaching 30% in 2019, but we were unable to decipher whether this increase occurred in community or healthcare settings due to a lack of medical records. However, as this setting is a surgical one serving a population of complicated inpatients and outpatients, this increase is more likely to occur in healthcare-associated infections. Indeed, this global trend was also recorded by the Laboratory of Antibio-Resistance in Tunisia (LART), the surveillance system of antimicrobial resistance data in clinical settings in Tunisia, as the rates of broad-spectrum cephalosporin resistance in *E. coli* jumped from 2.4% in 2000 to 18.8% in 2019 (16). However, accurate data about the prevalence of community-acquired ESBL EC are scarce in Tunisia but are likely to be high as the ESBL-EC carriage was found to be 19.63% among Tunisian healthy individuals (15).

The prevalence of ESBL-EC varies greatly throughout the world, ranging from approximately 5% to 20% in Europe, Australia, Canada, and the United States to >40% in other places like China, India, and South America. However, the prevalence of ESBL-EC is increasing worldwide. Importantly, the United States of America reported a 50% increase in hospital- and community-acquired infections due to ESBL producers between 2012 and 2017 (17).

The significant increase in the incidence of HA ESBL-EC identified in our study reaching 32 cases per 100,000 patient days in 2019 is consistent with previous studies demonstrating a rising rate of ESBL-EC in American tertiary care hospitals ranging from 11.1 infections per 100,000 patient days in 2009 to 22.1 infections per 100,000 patient days in 2014 (18) and in a French hospital where ESBL-EC incidence increased from 1.8 cases per 100,000 patient days in the year 2000 to 50 cases per 100,000 patient days in 2014 (19).

The present study confirms previous data on ESBL epidemiology in our hospital, in Tunisia, and worldwide (8, 10). CTX-Ms are largely predominant. CTX-M-15 is the most prevalent worldwide, in particular, because of its successful association with *E. coli* ST131. It was found in 57% of our isolates, and its progress continued during the study period. The CTX-M-27 variant (15.5%) remains the second variant in terms of prevalence after CTX-M-15. It is currently emerging and described in many countries such as France (20), in particular within ST131 (in clade A, in clade C and, in particular, in subclade C1-M27) (9, 20). Conversely, non-ST131-ESBL-E isolates showed a wide variety of ESBL types.

In a previous study conducted in our hospital, we demonstrated that ST131 accounted for 14.7% of ESBL-EC isolated between 1989 and 2009 (10). In this study, we described the continuous increase in ST131 prevalence that accounted for 42.3% of all HA ESBL-E between 2001 and 2009. This is in line with recent surveillance studies showing that its overall prevalence ranges from 12.5% to 30% of all *E. coli* clinical isolates and from 50% to 60% of ESBL-producing isolates (1, 6). In accordance with many studies, our nosocomial ST131 population was dominated by clade C isolates (>98%), with 66% belonging to clade C2 (all producing CTX-M-15) and 27% to C1 clade (all producing CTX-M-27) (9, 21, 22). Indeed, Merino et al. showed recently that the C1-M27 is responsible for 27% of 144 clinical ST131 obtained from different sites in Europe (23). Interestingly, our study period captured the emergence and expansion of ST131 lineages in our setting. The introduction of the C2 lineage in 2003 only disturbed the population structure transiently, after which it quickly reached a new equilibrium, and subsequently, ST131 maintained a stable proportion of the population around 20%. Likewise, the introduction of the C1 lineage in 2013 disturbed again the ESBL-E population structure, and subsequently, ST131 succeeded in becoming dominant in the whole population at around 60%. These findings are consistent with previous studies, which demonstrated that cluster C1-M27 isolates, first described in Japan (9), are now expanding in many other countries such as Thailand, Australia, Canada, USA, France, Italy, Germany, The Netherlands, and Spain (8, 9, 20, 22–24).

Although the C2 subclade isolates showed a higher virulence score (mean 15.94) compared with the subclade C1- M27 ST131 isolates (mean 13.90) (*P* < 0.001) and non-ST131 isolates (mean 6.44) (*P* < 0.001) and also the highest resistance rates to antibiotics (Table 1), the recent success and expansion of the C1-M27 subclades are likely related to its better colonizing abilities and higher transmission rates as demonstrated previously (25, 26). Moreover, we previously showed the clonal spread of many ST131-C1-M27 isolates among food handlers sharing the same workplace in Tunisia (15).

It is also noteworthy that our study has compared the ESBL-E population structure of HA and CA isolates. So far, very few studies have compared HA- and CA-ESBL-EC isolated in the same setting. Although ST131 prevalence was significantly higher in CA isolates, the phylogroup B2 accounted for the largest subset of ESBL-E causing both CA and HA infection in our setting. Unexpectedly, less than 9% (8.7% NA, 8.1% CA) of the ESBL-producing phylogroup B2 isolates in our collections was non-ST131. This is in contrast to recent studies demonstrating that historical clones ST73 and ST 95 may account for more than 40% of B2 isolates (27, 28). Moreover, although the global ST1193 prevalence has been increasing since 2012, even replacing ST131 in certain regions, this clone has been detected in only two HA CTX-M-15 ESBL-E isolates in 2011 and 2017. During the study period, ST131 remained the only phylogroup B2 clone that successfully evolved by acquiring plasmids harboring *bla*CTX-M-15 and *bla*CTX-M-27 genes and diversifying its clades with enrichment in VFs. It is also noteworthy that the distribution of the ST131 subclones with the predominance of C2-M15 clone and the recent emergence of C1-M27 clone observed in the HA-ESBL collection mirrors the ST131 epidemiology observed among our CA-ESBL collection and also that of the ESBL-E collected among healthy carriage in the same setting. This indicates that the *E. coli* causing HA infections do not form a different population from the community one but represent a spill-over of *E. coli* that colonizes the commensal niche in the wider human population (20). Importantly, both ST131 and non-ST131 ESBL-E isolates acquired in the community harbored significantly more VFs than those acquired in the hospital setting. Even C2 isolates were more virulent in the community than in the hospital. Importantly, as ESBL-producing ST131 displayed a large distinct and variable virulence content (Fig. 3), ST131 should not be considered as a unified clone but a cluster of distinct clonal subsets under ongoing diversification. Aside from *bla*_ESBL_, we detected significantly higher antibiotic resistance rates in nosocomial non-ST131 isolates compared with community non-ST131 isolates with the alarming emergence of carbapenemase, *mcr*, and *fosA* genes in the nosocomial setting representing a challenge for the treatment of these infections.

The study period captured the emergence of carbapenemase genes in our *E. coli* population in 2010. In contrast to the trend observed for ESBL-EC, the incidence of CP-EC remained low and stable over the study period with a highly diverse content in carbapenemase genes dominated by OXA-48-like genes which are prevalent in North Africa (29). Despite its huge success in ESBL-EC, the ST131 lineage failed to become dominant in the whole CP-EC population as it accounted for only 16% of this population. In fact, Huang et al. demonstrated that the CPE isolates exhibit diverse spatiotemporal epidemiological characteristics across the globe dominated by phylogroup A with a recent transition in the prevailing STs from highly virulent ST131 and ST38 to higher antibiotic-resistant ST410 and ST167 (30). Although the CP-ECs were not as virulent as the ESBL-EC in our collection, the emergence of highly resistant strains coproducing *bla*_OXA-48_ and *mcr-1* genes in Tunisia is worrisome and requires ongoing genomic surveillance.

Finally, the main change with respect to the previous study conducted at the Habib Bourguiba Hospital is the emergence of CTX-M27 and carbapenemase among ESBL-EC (10). Although previous Tunisian studies have reported the predominance of *bla*_CTX-M-15_ and particularly the CTX-M-15-C2 subclade among clinical ESBL-EC (11, 12, 31), this is the first report to demonstrate the recent expansion of the CTX-M-27-C1 subclone in both hospital and community settings in Tunisia and corroborates our previous finding of its dissemination among healthy carriers in the same setting (15). The findings of this study are also in line with currently available evidence, which supports the emergence of bla*_CTX_*_-*M*-27_ as a challenger for *bla*_CTX-M-15_ (8). Although this study includes long-term surveillance data, we acknowledge the presence of some limitations. First, we could not perform sequencing to better characterize the genomic structure of the studied *E. coli* strains. Second, we acknowledge a potential bias in the studied HA ESBL-EC as some strains were not stored. Third, the generalizability of our findings is impacted by the unicentric approach. However, this does not impact our main findings, as we conducted, to the best of our knowledge, the first large Tunisian study comparing over a long period of time both hospital-acquired and community-acquired ESBL-E.

### Conclusion

In conclusion, we described a steady increase in the prevalence of ESBL-EC and the emergence of CP-EC causing HA infections in a Tunisian hospital over a 19-year period. ST131 was the most frequent lineage identified among ESBL-EC, mainly linked to the expansion of the CTX-M-15-C2 subclade and recently the CTX-M-27-C1 subclade, both in hospital and in community settings. Future genomic surveillance studies are needed to better understand the expansion dynamics and structure of these successful clones and to guide the prevention strategies of their spread.

## MATERIALS AND METHODS

### Study design and *E. coli* isolates

The present study included all clinically relevant *E. coli* isolated in the Laboratory of Microbiology of HBUH of Sfax, Tunisia, between 2001 and 2019. Using the laboratory database, we selected all non-duplicate ESBL-EC and CP-E (first isolate within 30 days) associated with HA infections that had been stored in the laboratory archive. HA *E. coli* infections were defined as cases in which *E. coli* cultures were obtained from inpatients after at least 48 hours of hospital admission (32). HBUH has approximately 500 acute-care beds and includes principally surgical wards with intensive care units. The incidence of HA ESBL-EC and CP-E was expressed as the number of new cases per 1,000 patient days.

The study also included a collection of 107 ESBL-EC isolated from community-acquired infections diagnosed between 2011 and 2016 in two primary care outpatient clinics in Sfax, Tunisia. These CA ESBL-EC were isolated from outpatients without any previous contact with the healthcare service during the last year (32).

### Antibiotic susceptibility testing and ESBL typing

Antimicrobial susceptibility testing was performed according to the European Committee on Antimicrobial Susceptibility Testing standards using the disk diffusion method, E-test, or broth microdilution when recommended (33). ESBL and carbapenemase production were screened by the double-disc synergy and the carbapenem inactivation method tests, respectively (34, 35).

### Detection of resistance genes

Genes encoding ESBLs (*bla*_TEM_, *bla*_SHV_, and *bla*_CTX-M_) and carbapenemases (*bla*_OXA-48-like_, *bla*_NDM_, *bla*_VIM_, *bla*_KPC_, *bla*_IMP_, and *bla*_GES_) were characterized by PCR and sequencing (36). The presence of plasmid-mediated quinolone resistance determinants [*qnrA*, *qnrB*, *qnrC*, *qnrD*, *qnrS*, and *aac(6′)-Ib-cr*], *fosA,* and *mcr* genes were detected by PCR as previously described (37–39).

### Molecular characterization of *E. coli* isolates

The determination of the phylogenetic groups was carried out by the protocol of Clermont et al. (40). The ST131 clonal group and O16/O25b variants were identified as previously described (41). The *H*30Rx subclone was established by PCR detection of a specific SNP (G723A) of *ybbW* gene (6). The ST131 clades (A, B, and C), subclade C2, and the two clusters of subclade C1 (C1-M27 and the non-C1-M27) were established using the PCR assay developed by Matsumura et al. (42).

The presence of 26 VF genes and 9 plasmid addiction systems (PAS) were screened by PCR (7, 10, 15, 43). The virulence gene score was calculated as the number of VF genes detected. Isolates were classified as extraintestinal pathogenic *E. coli* (ExPEC) if positive for ≥2 of five VF genes markers, including *papAH* and/or *papC*, *sfa/focDE*, *afa/draBC*, and *kpsM II* (43). The virotypes A to F of the ST131 isolates were assigned according to the scheme developed by Dahbi et al. (7). A heatmap showing the ST131 VF and PAS profiles was constructed using the pheatmap package in R4.3.1.

### Statistical analysis

Pearson’s chi-square test or Fisher’s exact test and the Mann-Whitney U test were used for comparisons involving categorical and continuous variables, respectively. A *P* value < 0.05 was considered statistically significant. Statistical analyses were carried out using R (version 4.3.1).

## SUPPLEMENTAL MATERIAL

Supplemental tables spectrum.01471-24-s0001.docx

ASM does not own the copyrights to Supplemental Material that may be linked to, or accessed through, an article. The authors have granted ASM a non-exclusive, world-wide license to publish the Supplemental Material files. Please contact the corresponding author directly for reuse.
