# Supplementary material for: Extended-spectrum beta-lactamase- and carbapenemase-producing Escherichia coli isolates causing hospital- and community-acquired infections in Tunisia (2001–2019): expansion of CTX-M-15-C2 and CTX-M-27-C1 ST131 subclades
Source: Microbiol Spectr. 2024 Oct 25;12(12):e01471-24. doi: 10.1128/spectrum.01471-24 (PMC11619393; doi:10.1128/spectrum.01471-24)
Supplement: Supplemental tables [file spectrum.01471-24-s0001.docx]

Table S1: Distribution of hospital-acquired ESBL-producing *E. coli* isolates among wards and clinical specimens.

|  | Wards |  |  |  |  |  |  |  | Specimens | |  |  |  |  |  |  |
| --- | --- | --- | --- | --- | --- | --- | --- | --- | --- | --- | --- | --- | --- | --- | --- | --- |
|  | ICUs | URO | SURG | ORT | NCH | CV | MED | ENT | Urine | Blood | Resp | ABD | O_fluids | Wounds | Ca | TOTAL |
| 2019 | 10 | 9 | 8 | 2 | 2 |  | 2 |  | 22 | 2 | 3 | 4 | 1 | 1 |  | 33 |
| 2018 | 11 | 10 | 9 | 3 | 1 | 5 | 2 |  | 24 | 2 | 2 | 6 | 3 | 3 | 1 | 41 |
| 2017 | 8 | 9 | 8 | 1 | 3 | 1 | 1 |  | 19 | 2 | 2 | 6 | 1 | 1 |  | 31 |
| 2016 | 7 | 9 | 7 |  |  |  |  |  | 10 | 3 | 2 | 7 |  |  | 1 | 23 |
| 2015 | 3 | 2 | 6 | 3 | 1 |  | 2 | 1 | 5 | 3 | 3 | 4 | 1 | 2 |  | 18 |
| 2014 | 4 | 7 | 4 |  |  | 2 | 2 | 1 | 9 | 1 | 2 | 4 |  | 4 |  | 20 |
| 2013 | 9 | 5 | 5 | 3 | 2 | 2 | 3 |  | 15 | 4 | 5 | 1 | 1 | 3 |  | 29 |
| 2012 | 7 | 6 | 9 |  |  |  | 1 |  | 9 | 1 | 6 | 7 |  |  |  | 23 |
| 2011 | 3 | 5 | 3 |  | 1 | 1 | 1 |  | 7 | 2 | 1 | 2 |  | 2 |  | 14 |
| 2010 | 3 | 7 | 2 |  |  |  | 3 | 2 | 13 | 1 | 2 | 1 |  |  |  | 17 |
| 2009 | 7 | 5 | 2 | 1 | 1 | 3 |  |  | 9 | 1 | 3 | 2 |  | 3 | 1 | 19 |
| 2008 | 3 | 4 | 1 |  | 1 | 1 |  |  | 6 | 2 |  | 1 |  | 1 |  | 10 |
| 2007 | 2 | 7 | 4 |  |  |  | 1 |  | 8 | 1 | 1 | 3 |  | 1 |  | 14 |
| 2006 | 6 | 7 | 1 |  |  |  | 1 | 1 | 9 | 3 | 1 |  |  | 2 | 1 | 16 |
| 2005 | 4 | 5 |  |  |  |  | 3 |  | 7 | 3 |  | 1 |  | 1 |  | 12 |
| 2004 | 9 | 3 |  | 1 | 1 |  | 4 |  | 7 | 4 | 3 | 1 |  | 3 |  | 18 |
| 2003 | 2 | 9 | 3 |  |  | 1 |  |  | 10 | 2 | 1 | 2 |  |  |  | 15 |
| 2002 | 1 |  | 5 |  |  |  |  |  | 2 |  | 1 | 1 |  | 2 |  | 6 |
| 2001 | 3 | 3 | 1 | 1 | 2 | 1 | 1 |  | 8 | 3 |  |  |  | 1 |  | 12 |
|  | 102 | 112 | 78 | 15 | 15 | 17 | 27 | 5 | 199 | 40 | 38 | 53 | 7 | 30 | 4 | 371 |

ICU: intensive care unit, URO: urology, SURG: General Surgery, ORT: Orthopaedics, NCH: Neurosurgery, Cardiothoracic surgery, ENT: Otolaryngology, Resp: respiratory fluids, ABD: Abdominal fluids, O_fluids: other fluids (including prosthetic joint infection and pleural effusion), Ca: catheter.

Table S2: Distribution of hospital-acquired carbapenemase-producing *E. coli* isolates among wards and clinical specimens.

|  | Wards |  |  |  |  |  |  |  | Specimens | |  |  |  |  |  |
| --- | --- | --- | --- | --- | --- | --- | --- | --- | --- | --- | --- | --- | --- | --- | --- |
|  | ICUs | URO | SURG | ORT | NCH | CV | MED | ENT | Urines | Blood | RESP | ABD | O_Fluids | Wounds | TOTAL |
| 2019 | 1 |  |  | 2 |  |  |  |  | 2 |  |  |  | 1 |  | 3 |
| 2018 | 1 | 1 | 2 |  |  | 1 |  |  | 2 |  |  | 2 |  | 1 | 5 |
| 2017 | 1 | 1 |  |  |  |  |  |  | 1 |  | 1 |  |  |  | 2 |
| 2016 | 2 | 1 | 1 | 2 |  |  |  |  | 2 |  | 1 | 1 | 1 | 1 | 6 |
| 2015 |  |  | 1 |  |  |  |  | 1 |  |  |  |  |  | 2 | 2 |
| 2014 |  |  |  | 1 |  | 1 | 1 |  |  |  |  |  | 1 | 2 | 3 |
| 2013 | 4 | 1 | 1 |  |  | 1 |  |  | 2 | 1 | 3 |  |  | 1 | 7 |
| 2012 |  |  | 3 | 1 |  |  |  |  | 1 |  |  | 2 |  | 1 | 4 |
| 2011 |  |  | 1 |  |  | 1 |  |  |  |  |  |  |  | 2 | 2 |
| 2010 | 1 | 1 | 1 |  |  |  |  |  | 2 |  | 1 |  |  |  | 3 |
|  | 10 | 5 | 10 | 6 | 0 | 4 | 1 | 1 | 12 | 1 | 6 | 5 | 3 | 10 | 37 |

ICU: intensive care unit, URO: urology, SURG: General Surgery, ORT: Orthopaedics, NCH: Neurosurgery, Cardiothoracic surgery, ENT: Otolaryngology, Resp: respiratory fluids, ABD: Abdominal fluids, O_fluids: other fluids (including prosthetic joint infection and pleural effusion), Ca: catheter.

Table S3: Molecular characteristics and antibiotic susceptibility of the carbapenemase-producing *E. coli* isolates

|  | **No. (%) of isolates** | | | | | | **P-value** | | |
| --- | --- | --- | --- | --- | --- | --- | --- | --- | --- |
|  | **HA ESBL**  **ST131**  **(151)** | **HA ESBL**  **Non-ST131**  **(201)** | **HA ESBL+**  **CP-EC ST131**  **(6)** | **HA ESBL+**  **CP-EC Non-ST131**  **(13)** | **HA**  **CP-EC**  **ST131**  **(0)** | **HA**  **CP-EC**  **Non-ST131**  **(18)** | **HA ESBL -EC ST131 vs. HA ESBL + CP-EC ST31** | **HA ESBL -EC Non-ST131 vs. HA ESBL + CP-EC Non-ST31** | **HA ESBL -EC Non-ST131 vs. HA CP-EC Non-ST31** |
|  |  |  |  |  |  |  |  |  |  |
|  |  |  |  |  |  |  |  |  |  |
| **Phylogroups** |  |  |  |  |  |  |  |  |  |
| A |  | 105 (52.2) |  | 9 (69.2) |  | 10 (55.5) |  |  |  |
| B1 |  | 31 (15.4) |  | 1 (7.7) |  | 3 (16.6) |  |  |  |
| D |  | 50 (24.8) |  | 3 (23) |  | 4 (22.2) |  |  |  |
| B2 | 151 (100) | 15 (7.4) | 6 (100) | 0 |  | 1 (5.5) |  |  |  |
| ST131-C2 | 98 (65) |  | 5 (83.3) |  |  |  |  |  |  |
| ST131 C1M27 | 42 (27.8) |  | 0 |  |  |  |  |  |  |
| ST131 C1nM27 | 8 (5.2) |  | 1 (16.6) |  |  |  |  |  |  |
| **Virulence factors** | | | | | | | | | |
| **Adhesin** |  |  |  |  |  |  |  |  |  |
| *fimH* | 151 (100) | 170 (84.5) | 6 (100) | 13 (100) |  | 15 (83.3) |  |  |  |
| *papAH* | 42 (27.8) | 33 (16.4) | 4 (66.6) | 5 (38.4) |  | 4 (22.2) |  |  |  |
| *papC* | 65 (43) | 24 (11.9) | 3 (50) | 1 (7.7) |  | 5 (27.7) |  |  |  |
| *papEF* | 33 (21.8) | 34 (16.9) | 3 (50) | 1 (7.7) |  | 3 (16.6) |  |  |  |
| *papGI* | 0 | 2 (0.9) | 0 | 0 |  | 0 |  |  |  |
| *papGII-III* | 41 (27.1) | 19 (9.4) | 4 (66.6) | 2 (15.4) |  | 0 |  |  |  |
| *sfa/focDE* | 0 | 31 (15.4) | 0 | 4 (30.7) |  | 1 (5.5) |  |  |  |
| *afa/draBC* | 6 (3.9) | 6 (2.9) | 1 (16.6) | 1 (7.7) |  | 1 (5.5) |  |  |  |
| *yfcV* | 151 (100) | 35 (17.4) | 6 (100) | 8 (61.5) |  | 4 (22.2) |  | 0.00048 |  |
| **Toxin** |  |  |  |  |  |  |  |  |  |
| *sat* | 145 (96) | 54 (26.8) | 2 (33.3) | 2 (15.4) |  | 5 (27.7) | <0.00001 |  |  |
| *cnf1* | 45 (29.8) | 12 (5.9) | 0 | 0 |  | 0 |  |  |  |
| *hlyA* | 45 (29.8) | 13 (6.4) | 4 (66.6) | 0 |  | 0 |  |  |  |
| **Iron uptake** |  |  |  |  |  |  |  |  |  |
| *iutA* | 148 (98) | 118 (58.7) | 6 (100) | 11 (84.6) |  | 11 (61.1) |  |  |  |
| *iroN* | 3 (1.9) | 31 (15.4) | 4 (66.6) | 2 (15.4) |  | 4 (22.2) | <0.00001 |  |  |
| *fyuA* | 151 (100) | 97 (48.2) | 5 (83.3) | 5 (38.4) |  | 10 (55.5) | 0.01568 |  |  |
| *chuA* | 151 (100) | 50 (24.8) | 6 (100) | 3 (23) |  | 5 (27.7) |  |  |  |
| **Capsule** |  |  |  |  |  |  |  |  |  |
| *kpsM II* | 147 (97.3) | 51 (25.3) | 6 (100) | 3 (23) |  | 5 (27.7) |  |  |  |
| *kpsM II-K5* | 88 (58.2) | 30 (14.9) | 3 (50) | 3 (23) |  | 3 (16.6) |  |  |  |
| *kpsM III* | 5 (3.3) | 20 (9.9) | 0 | 1 (7.7) |  | 0 |  |  |  |
| **Miscellaneous** |  |  |  |  |  |  |  |  |  |
| *irp2* | 150 (99.3) | 105 (52.2) | 6 (100) | 5 (38.4) |  | 11 (61.1) |  |  |  |
| *ear* | 145 (96) | 114 (56.7) | 3 (50) | 8 (61.5) |  | 13 (72.2) | 0.00011 |  |  |
| *cvaC* | 5 (3.3) | 11 (5.4) | 0 | 1 (7.7) |  | 4 (22.2) |  |  | 0.02723 |
| *hra* | 57 (37.7) | 43 (21.3) | 5 (83.3) | 4 (30.7) |  | 4 (22.2) |  |  |  |
| *pher* | 102 (67.5) | 147 (73.1) | 5 (83.3) | 10 (76.9) |  | 9 (50) |  |  |  |
| *traT* | 108 (71.5) | 140 (69.6) | 6 (100) | 9 (69.2) |  | 12 (66.6) |  |  |  |
| *ibeA* | 3 (1.9) | 3 (1.5) | 0 | 0 |  | 0 |  |  |  |
| *malX* | 151 (100) | 36 (17.9) | 6 (100) | 2 (15.4) |  | 3 (16.6) |  |  |  |
| *usp* | 151 (100) | 26 (12.9) | 6 (100) | 3 (23) |  | 0 |  |  |  |
| **Mean per isolate** | **15.1** | **7.2** | **16.6** | **8.2** | **0** | **7.3** |  |  |  |
| **ExPEC*** | 144 (95.3) | 65 (32.3) | 6 (100) | 7 (53.8) | 0 | 6 (33.3) |  |  |  |
| **Virotype** |  |  |  |  |  |  |  |  |  |
| A | 5 (3.3) |  | 1 (16.6) | - |  | - |  |  |  |
| B | 3 (2) |  | 0 | - |  | - |  |  |  |
| C | 88 (58.2) |  | 1( 16.6) | - |  | - |  |  |  |
| D | 3 (2) |  | 0 | - |  | - |  |  |  |
| E | 43 (28.4) |  | 0 | - |  | - |  |  |  |
| F | 3 (2) |  | 0 | - |  | - |  |  |  |
| IND | 6 (4) |  | 4 (66.6) | - |  | - | <0.00001 |  |  |
| **Resistance**  **genes** | | | | | | | | | |
| *bla*_CTX-M-15_ | 100 (66.2) | 135 (67.1) | 5 (83.3) | 11 (84.6) |  | - |  |  |  |
| *bla*_CTX-M-27_ | 49 (32.4) | 2 (1) | 1 (16.6) | 0 |  | - |  |  |  |
| *bla*_CTX-M-14_ | 0 | 22 (11) | 0 | 2 (15.3) |  | - |  |  |  |
| *bla*_CTX-M-1_ | 0 | 21 (10.4) | 0 | 0 |  | - |  |  |  |
| *bla*_CTX-M-55_ |  | 4 (2) |  |  |  |  |  |  |  |
| *bla*_SHV-12_ | 2 (1.3) | 20 (10) | 0 | 1 (7.7) |  | - |  |  |  |
| *bla*_SHV-2a_ | 0 | 6 (3) | 0 | 0 |  | - |  |  |  |
| *bla*_TEM-26_ | 0 | 1 (0.5) | 0 | 0 |  | - |  |  |  |
| *bla*_OXA-48_ | 0 | 0 | 1 (16.6) | 7 (53.8) |  | 13 (72.2) | 0.01568 | <0.00001 | <0.00001 |
| *bla*_OXA-204_ | 0 | 0 | 3 (50) | 1 (7.7) |  | 2 (11) | <0.00001 |  | 0.000551 |
| *bla*_OXA-244_ |  | 0 | 0 | 0 |  | 2 (11) |  |  | 0.000551 |
| *bla*_OXA-181_ | 0 | 0 | 0 | 1 (7.7) |  | 1 (5.5) |  |  |  |
| *bla*_NDM-1_ | 0 | 0 | 2 (33.3) | 3 (23) |  | 0 | <0.00001 | <0.00001 |  |
| *bla*_VIM-2_ | 0 | 0 | 0 | 1 (7.7) |  | 0 |  |  |  |
| *aac(6’)-Ib-cr* | 98 (65) | 90 (44.7) | 5 (83.3) | 9 (69.2) |  | 0 |  |  | 0.000562 |
| *qnrA* | 2 (1.3) | 2 (1) | 0 | 0 |  | 0 |  |  |  |
| *qnrB* | 1 (0.6) | 9 (4.4) | 0 | 2 (15.3) |  | 1 (5.5) |  |  |  |
| *qnrS* | 0 | 7 (3.5) | 0 | 1 (7.7) |  | 1 (5.5) |  |  |  |
| *aqxAB* | 0 | 2 (1) | 0 | 0 |  | 0 |  |  |  |
| *mcr-1* | 0 | 2 (1) | 0 | 0 |  | 0 |  |  |  |
| *fosA3* | 0 | 0 | 0 | 0 |  | 0 |  |  |  |
| **Antibiotics** |  |  |  |  |  |  |  |  |  |
| Amoxicillin-clavulanate | 103 (68.2) | 158 (78.6) | 6 (100) | 13 (100) |  | 18 (100) |  |  | 0.06021 |
| imipenem | 3 (2) | 2 (1) | 3 (50) | 11 (84.6) |  | 15 (83.3) | <0.00001 | <0.00001 | <0.00001 |
| Amikacin | 33 (21.8) | 27 (13.4) | 1 (16.6) | 3 (23) |  | 1 (5.5) |  |  |  |
| Gentamicin | 73 (48.3) | 131 (65.1) | 5 (83.3) | 11 (84.6) |  | 7 (38.8) |  |  | 0.05021 |
| Tobramycin | 96 (63.5) | 149 (74.1) | 5 (83.3) | 11 (84.6) |  | 8 (44.4) |  |  | 0.01617 |
| Ciprofloxacin | 149 (98.6) | 151 (75.1) | 6 (100) | 11 (84.6) |  | 12 (66.6) |  |  |  |
| Trimethoprim/ sulfamethoxazole | 112 (74) | 149 (74.1) | 6 (100) | 10 (77) |  | 9 (50) |  |  |  |
| Fosfomycin | 1 (0.6) | 3 (1.5) | 1 (16.6) | 0 |  | 2 (11) |  |  |  |
| **Addiction** **systems** | | | | | | | | | |
| *ccdAB* | 138 (91.4) | 129 (64.1) | 6 (100) | 7 (53.8) |  | 8 (44.4) |  |  |  |
| *pemKI* | 131 (86.7) | 144 (71.6) | 6 (100) | 10 (77) |  | 18 (100) |  |  | 0.01895 |
| *srnCB* | 109 (72) | 120 (59.7) | 4 (66.6) | 8 (61.5) |  | 9 (50) |  |  |  |
| *hok/sok* | 40 (26.5) | 64 (31.8) | 2 (33.3) | 1 (7.7) |  | 8 (44.4) |  |  |  |
| *pndAC* | 16 (10.6) | 22 (11) | 1 (16.6) | 2 (15.3) |  | 2 (11) |  |  |  |
| *vagCD* | 99 (65.5) | 103 ( 51.2) | 6 (100) | 7 (53.8) |  | 3 (16.6) |  |  | 0.01029 |
| *yacAB* | 18 (12) | 42 (20.9) | 0 | 3 (23) |  | 7 (38.8) |  |  |  |
| *relEB* | 3 (2) | 5 (2.4) | 0 | 0 |  | 2 (11) |  |  |  |
| **Mean per isolate** | **3.6** | **3.1** | **4.1** | **2.9** |  | **3.1** | 0 |  |  |

^*^: Isolates were categorized as extra-intestinal pathogenic *E. coli* (ExPEC) if more than 2 of 5 virulence factors, including *papAH* and/or *papC*, sfa/*focDE*, afa/*draBC*, *kpsM* *II*, and *iutA* were detected

Table S4: Molecular characteristics and antibiotic susceptibility of the ESBL-*E. coli* isolates according to community or hospital origin

|  | **No. (%) of isolates** | | | | | | **P-value** | | |
| --- | --- | --- | --- | --- | --- | --- | --- | --- | --- |
|  | **HA ESBL**  **ST131**  **(157)** | **HA ESBL**  **ST131 C2**  **(103)** | **HA ESBL**  **Non-ST131**  **(214)** | **CA ESBL**  **ST131**  **(68)** | **CA ESBL**  **ST131 C2**  **(50)** | **CA ESBL**  **Non-ST131**  **(39)** | **HA vs. CA ESBL**  **ST131** | **HA vs. CA ESBL Non-ST131** | **HA vs. CA**  **ESBL**  **ST131 C2** |
|  |  |  |  |  |  |  |  |  |  |
|  |  |  |  |  |  |  |  |  |  |
| **Phylogroups** |  |  |  |  |  |  |  |  |  |
| A |  |  | 114 (53.3) |  |  | 12 (30.7) |  | 0.01592 |  |
| B1 |  |  | 32 (14.9) |  |  | 5 (12.8) |  |  |  |
| D |  |  | 53 (24.8) |  |  | 16 (41) |  | 0.05725 |  |
| B2 | 157 (100) |  |  | 68 (100) |  | 6 (15.4) |  |  |  |
| ST131-C2 | 103 (65.6) |  |  | 50 (73.5) |  |  |  |  |  |
| ST131 C1M27 | 42 (26.7) |  |  | 8 (11.8) |  |  | 0.02097 |  |  |
| ST131 C1nM27 | 9 (5.7) |  |  | 8 (11.8) |  |  |  |  |  |
| **Virulence factors** |  |  |  |  |  |  |  |  |  |
| **Adhesin** |  |  |  |  |  |  |  |  |  |
| *fimH* | 157 (100) | 103 (100) | 183 (85.5) | 68 (100) | 50 (100) | 28 (71.7) |  |  |  |
| *papAH* | 46 (29.3) | 45 (43.7) | 38 (17.8) | 40 (58.8) | 39 (78) | 11 (28.2) | <0.00001 |  | 0.00012 |
| *papC* | 68 (43.3) | 42 (40.8) | 25 (11.7) | 52 (76.4) | 43 (86) | 13 (33.3) | <0.00001 | 0.00120 | 0.00120 |
| *papEF* | 36 (22.9) | 36 (35) | 35 (16.4) | 33 (48.5) | 32 (64) | 12 (30.7) | <0.00001 |  | 0.00129 |
| *papGI* | 0 | 0 | 2 (0.9) | 0 | 0 | 0 |  |  |  |
| *papGII-III* | 45 (28.7) | 45 (43.7) | 21 (9.8) | 47 (69.1) | 43 (86) | 17 (43.6) | <0.00001 | <0.00001 | <0.00001 |
| *sfa/focDE* | 0 | 0 | 35 (16.4) | 1 (1.4) | 1 (2) | 3 (7.7) |  |  |  |
| *afa/draBC* | 7 (4.5) | 7 (6.8) | 7 (3.3) | 1 (1.4) | 5 (10) | 3 (7.7) |  |  |  |
| *yfcV* | 157 (100) | 103 (100) | 43 (20.1) | 1 (1.4) | 50 (100) | 9 (23) | <0.00001 |  |  |
| **Toxin** |  |  |  |  |  |  |  |  |  |
| *sat* | 147 (93.6) | 97 (94.2) | 56 (26.2) | 68 (100) | 50 (100) | 9 (23) |  |  |  |
| *cnf1* | 45 (28.7) | 45 (43.7) | 12 (5.6) | 42 (61.7) | 41 (82) | 6 (15.4) | <0.00001 | 0.06492 | <0.00001 |
| *hlyA* | 49 (31.2) | 49 (47.6) | 13 (6.1) | 41 (61.7) | 40 (80) | 8 (20.5) | <0.00001 | 0.00714 | 0.00027 |
| **Iron uptake** |  |  |  |  |  |  |  |  |  |
| *iutA* | 154 (98.1) | 103 (100) | 129 (60.3) | 66 (97) | 48 (96) | 26 (66.6) |  |  |  |
| *iroN* | 7 (4.5) | 7 (6.8) | 33 (15.4) | 2 (2.9) | 0 | 4 (10.2) |  |  |  |
| *fyuA* | 157 (100) | 103 (100) | 102 (47.7) | 68 (100) | 50 (100) | 26 (66.6) |  | 0.04454 |  |
| *chuA* | 157 (100) | 103 (100) | 53 (24.8) | 68 (100) | 50 (100) | 16 (41) |  |  |  |
| **Capsule** |  |  |  |  |  |  |  |  |  |
| *kpsM II* | 153 (97.5) | 99 (96.1) | 54 (25.2) | 66 (97) | 45 (90) | 9 (23) |  |  |  |
| *kpsM II-K5* | 91 (58) | 42 (40.8) | 33 (15.4) | 23 (33.8) | 13 (26) | 8 (20.5) | 0.00147 |  |  |
| *kpsM III* | 5 (3.2) | 5 (4.9) | 21 (9.8) | 1 (1.4) | 0 | 3 (7.7) |  |  |  |
| **Miscellaneous** |  |  |  |  |  |  |  |  |  |
| *irp2* | 156 (99.4) | 103 (100) | 110 (51.4) | 62 (91.2) | 45 (90) | 28 (71.8) | 0.00465 | 0.02945 | 0.00546 |
| *ear* | 148 (94.3) | 97 (94.2) | 122 (57) | 65 (95.6) | 50 (100) | 23 (59) |  |  |  |
| *cvaC* | 5 (3.2) | 4 (3.9) | 12 (5.6) | 0 | 0 | 0 |  |  |  |
| *hra* | 62 (39.5) | 56 (54.4) | 47 (22) | 46 (67.6) | 38 (76) | 16 (41) | <0.00001 | 0.01978 | 0.01634 |
| *pher* | 107 (68.2) | 55 (53.4) | 157 (73.4) | 46 (67.6) | 30 (60) | 28 (71.8) |  |  |  |
| *traT* | 114 (72.6) | 86 (83.5) | 149 (69.6) | 60 (88.2) | 47 (94) | 29 (74.3) | 0.01652 |  |  |
| *ibeA* | 3 (1.9) | 1 (1) | 3 (1.4) | 0 | 0 | 0 |  |  |  |
| *malX* | 157 (100) | 103 (100) | 38 (17.8) | 68 (100) | 50 (100) | 15 (38.4) |  | 0.00676 |  |
| *usp* | 157 (100) | 103 (100) | 29 (13.6) | 65 (95.6) | 50 (100) | 8 (20.5) | 0.04373 |  |  |
| **Mean per isolate** | 15.2 | 15.94 | 6.44 | 16.1 | 18.2 | 9.1 | <0.00001 | <0.00001 | <0.00001 |
| **ExPEC*** | 152 (96.8) | 101 (98.1) | 58 (27.1) | 66 (97) | 50 (100) | 14 (35.9) |  |  |  |
| **Virotype** |  |  |  |  |  |  |  |  |  |
| A | 6 (3.8) | 6 (5.8) |  | 2 (3) | 2 (4) | - |  |  |  |
| B | 3 (1.9) | 3 (2.9) |  | 2 (3) | 0 | - |  |  |  |
| C | 89 (56.7) | 40 (38.8) |  | 18 (26.4) | 5 (10) | - | <0.00001 |  | 0.00049 |
| D | 3 (1.9) | 1 (1) |  | 0 | 0 | - |  |  |  |
| E | 43 (27.4) | 43 (41.7) |  | 41 (60.2) | 41 (82) | - | <0.00001 |  | <0.00001 |
| F | 3 (1.9) | 3 (2.9) |  | 3 (4.4) | 1 (2) | - |  |  |  |
| IND | 10 (6.4) | 7 (6.8) |  | 2 (3) | 1 (2) | - |  |  |  |
| **Resistance** **genes** |  |  |  |  |  |  |  |  |  |
| *bla*_CTX-M-15_ | 105 (66.9) | 103 (100) | 142 (66.4) | 52 (76.4) | 50 (100) | 22 (56.4) |  |  |  |
| *bla*_CTX-M-27_ | 50 (31.8) | 0 | 4 (1.8) | 16 (23.5) | 0 | 4 (10.2) |  | 0.02411 |  |
| *bla*_CTX-M-14_ | 0 | 0 | 24 (11.2) | 0 | 0 | 8 (20.5) |  |  |  |
| *bla*_CTX-M-1_ | 0 | 0 | 21 (9.8) | 0 | 0 | 4 (10.2) |  |  |  |
| *bla*_CTX-M-55_ | 0 | 0 | 4 (1.8) | 0 | 0 | 0 |  |  |  |
| *bla*_SHV-12_ | 5 (3.2) | 5 (4.9) | 18 (8.4) | 4 (5.8) | 3 (6) | 3 (7.7) |  |  |  |
| *bla*_SHV-2a_ | 0 | 0 | 6 (2.8) | 0 | 0 | 0 |  |  |  |
| *bla*_TEM-26_ | 0 | 0 | 1 (0.5) | 0 | 0 | 0 |  |  |  |
| *bla*_OXA-48_ | 1 (0.6) | 1 (1) | 7 (3.3) | 0 | 0 | 0 |  |  |  |
| *bla*_OXA-204_ | 3 (1.9) | 3 (2.9) | 1 (0.5) | 0 | 0 | 0 |  |  |  |
| *bla*_OXA-181_ | 0 | 0 | 1 (0.5) | 0 | 0 | 0 |  |  |  |
| *bla*_NDM-1_ | 2 (1.3) | 1 (1) | 3 (1.4) | 0 | 0 | 0 |  |  |  |
| *bla*_VIM-2_ | 0 | 0 | 1 (0.5) | 0 | 0 | 0 |  |  |  |
| *aac(6’)-Ib-cr* | 103 (65.6) | 102 (99) | 99 (46.3) | 49 (72) | 48 (96) | 4 (10.2) |  | <0.00001 |  |
| *qnrA* | 2 (1.3) | 0 | 2 (0.9) | 0 | 0 | 0 |  |  |  |
| *qnrB* | 1 (0.6) | 0 | 11 (5.1) | 0 | 0 | 0 |  |  |  |
| *qnrS* | 0 | 0 | 8 (3.7) | 0 | 0 | 2 (5.1) |  |  |  |
| *aqxAB* | 0 | 0 | 2 (0.9) | 0 | 0 | 0 |  |  |  |
| *mcr-1* | 0 | 0 | 2 (0.9) | 0 | 0 | 0 |  |  |  |
| *fosA3* | 0 | 0 | 1 (0.5) | 0 | 0 | 0 |  |  |  |
| **Antibiotics** |  |  |  |  |  |  |  |  |  |
| Amoxicillin-clavulanate | 109 (69.4) | 95 (92.2) | 171 (79.9) | 54 (79.4) | 38 (76) | 28 (71.8) |  |  | 0.01114 |
| imipenem | 6 (3.8) | 5 (4.9) | 13 (6.1) | 0 | 0 | 0 |  |  |  |
| Amikacin | 34 (21.7) | 32 (31.1) | 30 (14) | 11 (16.1) | 10 (20) | 1 (2.5) |  |  |  |
| Gentamicin | 78 (49.7) | 75 (72.8) | 142 (66.4) | 42 (61.7) | 27 (54) | 12 (30.7) |  | <0.00001 | 0.03294 |
| Tobramycin | 101 (64.3) | 97 (94.2) | 160 (74.8) | 51 (75) | 37 (74) | 13 (33.3) |  | <0.00001 | 0.00101 |
| Ciprofloxacin | 155 (98.7) | 103 (100) | 162 (75.7) | 67 (98.5) | 50 (100) | 15 (38.4) |  | <0.00001 |  |
| Trimethoprim/ sulfamethoxazole | 118 (75.2) | 73 (70.9) | 159 (74.3) | 50 (73.5) | 38 (76) | 23 (59) |  |  |  |
| Fosfomycin | 2 (1.3) | 0 | 3 (1.4) | 0 | 0 | 0 |  |  |  |
| **Addiction** **systems** |  |  |  |  |  |  |  |  |  |
| *ccdAB* | 144 (91.7) | 90 (87.4) | 136 (63.6) | 67 (98.5) | 50 (100) | 22 (56.4) |  |  | 0.0205 |
| *pemKI* | 137 (87.3) | 85 (82.5) | 154 (72) | 65 (95.5) | 48 (96) | 18 (46) |  | 0.00278 | 0.03905 |
| *srnCB* | 113 (72) | 69 (67) | 128 (59.8) | 57 (83.8) | 41 (82) | 20 (51.2) |  |  |  |
| *hok/sok* | 42 (26.8) | 31 (30.1) | 65 (30.4) | 10 (14.7) | 6 (12) | 14 (35.9) | 0.07247 |  | 0.0244 |
| *pndAC* | 17 (10.8) | 15 (14.6) | 24 (11.2) | 5 (7.3) | 4 (8) | 7 (18) |  |  |  |
| *vagCD* | 105 (66.9) | 102 (99) | 110 (51.4) | 45 (66) | 45 (90) | 6 (15.3) |  | <0.00001 | 0.02415 |
| *yacAB* | 18 (11.5) | 11 (10.7) | 45 (21) | 3 (4.4) | 1 (2) | 12 (30.7) |  |  |  |
| *relEB* | 3 (1.9) | 3 (2.9) | 5 (2.3) | 0 | 0 | 4 (10.2) |  | 0.04705 |  |
| **Mean per isolate** | 3.69 | 3.94 | 3.12 | 3.7 |  | 2.6 |  | 0.049 |  |

^*^: Isolates were categorized as extra-intestinal pathogenic *E. coli* (ExPEC) if more than 2 of 5 virulence factors, including *papAH* and/or *papC*, sfa/*fo*
